# Twenty five years of the Training Program in Epidemiology Applied to the Unified Health System Services: contributions of field epidemiology to health surveillance in Brazil

**DOI:** 10.1590/S2237-96222026v35e20263002.en

**Published:** 2026-05-12

**Authors:** Mariana Del Grossi Moura, Taís Freire Galvão, Maria Auxiliadora Parreiras Martins, Everton Nunes da Silva, Jorge Otávio Maia Barreto

**Affiliations:** 1 Universidade de Sorocaba, Programa de Pós-graduação em Ciências Farmacêuticas, Sorocaba, SP, Brasil Universidade de Sorocaba Universidade de Sorocaba Programa de Pós-graduação em Ciências Farmacêuticas Sorocaba SP Brazil; 2 Universidade Estadual de Campinas, Faculdade de Ciências Farmacêuticas, Campinas, SP, Brasil Universidade Estadual de Campinas Universidade Estadual de Campinas Faculdade de Ciências Farmacêuticas Campinas SP Brazil; 3 Universidade Federal de Minas Gerais, Faculdade de Farmácia, Belo Horizonte, MG, Brasil Universidade Federal de Minas Gerais Universidade Federal de Minas Gerais Faculdade de Farmácia Belo Horizonte MG Brazil; 4 Universidade de Brasília, Faculdade de Ciências e Tecnologias em Saúde, Brasília, DF, Brasil Universidade de Brasília Universidade de Brasília Faculdade de Ciências e Tecnologias em Saúde Brasília DF Brazil; 5 Fundação Oswaldo Cruz, Gerência Regional de Brasília, Brasília, DF, Brasil Fundação Oswaldo Cruz Fundação Oswaldo Cruz Gerência Regional de Brasília Brasília DF Brazil

Health surveillance is a cornerstone of the Brazilian Unified Health System (*Sistema Único de Saúde*, SUS), acting as a cross-sectional and guiding piece of the care model. It involves collecting, analyzing, and disseminating information on the overall health landscape, in order to plan and implement actions for promotion, protection, and prevention, as well as the control of risks, diseases, and injuries ^1^. Given this context, the training of professionals to work in the health care services integrates the actions necessary for the development and organization of these activities in the SUS.

Therefore, the Training Program in Epidemiology Applied to the Unified Health System Services (*Programa de Treinamento em Epidemiologia Aplicada aos Serviços do Sistema Único de Saúde*, EpiSUS), of the Secretariat of Health and Environmental Surveillance, of the Brazilian Ministry of Health, is an in-service training strategy, emphasizing the application of epidemiological methods in the daily routine of health services ^2^. Structured in different levels of training, it seeks to develop one’s skills in detecting, investigating and responding to events of public health interest, integrating practical activities and technical oversight. Since its implementation, EpiSUS has contributed to the qualification of SUS professionals and to the fulfilment of field investigations and epidemiological analyses aimed at the demands of health surveillance ^2^.

In celebration of the 25^th^ anniversary of EpiSUS, in 2025, the *Epidemiologia e Serviços de Saúde: revista do SUS* (RESS) opened a call for the publication of field research prepared within the context of EpiSUS with the support of the Department of Public Health Emergencies of the Secretariat of Health and Environmental Surveillance. In addition to celebrating the trajectory of the program, RESS sought to contribute to the dissemination of evidence resulting from the investigations conducted by its former students. As a result, 12 original articles were published. These articles reflect relevant contributions to the SUS in the last decade, and are the object of this editorial.

In the first article, the authors report the experience of implementing EpiSUS between 2000 and 2025, presenting the historical milestones and expansion of training levels, reporting the qualification of more than 4,000 professionals in field epidemiology ^2^.

Investigation of outbreaks and other acute epidemiological events, remarkable motivators for the establishment of EpiSUS, were portrayed in different approaches. A retrospective cohort study described an outbreak of infections by *Mycobacterium abscessus* associated with cosmetic surgeries in the border between Brazil and Paraguay, from 2021 to 2022. Individuals who undertook lipoabdominoplasty between December, 2021 and January, 2022, had the highest risk of infection^3^. The report of an outbreak of chickenpox in Indigenous territory in the state of Bahia in 2024 identified vaccination coverage below the recommended target and described the control measures adopted, including vaccination updates and active case search ^4^. An article described the clinical-epidemiological profile and exposures of the first autochthonous cases of Oropouche fever in Southern Brazil, between April and June 2024. It is noteworthy that this was a previously non-endemic area, with no history of travel among individuals to other federative units or abroad, in addition to identifying environmental exposures compatible with vector transmission, such as waterbodies and areas with natural vegetation ^5^. 

An outbreak of foodborne hepatitis A that occurred in Curitiba between 2023 and 2024 resulted in five studies that elucidated different aspects of the field investigation. A case-control study identified a higher chance of hepatitis A associated with men who have sex with men and the consumption of raw fish, while eating at home was a protective factor ^6^. Among adolescents, a low seroprevalence of long-acting antibodies against hepatitis A was observed, with a predominance of vaccine immunity ^7^. This same population presented poor knowledge on hepatitis A, especially regarding transmission and symptoms, as well as risky sexual behaviors^8^. In people experiencing homelessness, a case series observed a higher frequency of severe forms of the infection and higher lethality compared to the general population, evincing the vulnerability of this group in the context of the outbreak ^9^. Based on another case series, the authors described the occurrence of severe forms of hepatitis A, including deaths and the need for liver transplantation, in addition to relevant clinical complications and the need for intensive support ^10^.

Studies on immunizations are part of the topics addressed in this issue. A cross-sectional study conducted in São Sebastião, Federal District, estimated a 54% vaccination coverage against yellow fever, lower than the target established by the National Immunization Program. The study also described the knowledge, attitudes, and practices of the local population, and it was identified that most had inadequate knowledge about vaccination against yellow fever, but with a higher frequency of appropriate attitudes and practices ^11^. 

An analysis of events supposedly attributable to vaccination-based on causality-identified an association between serious adverse events and characteristics such as batch, presentation, and laboratory producing the pentavalent vaccine (which protects against diphtheria, tetanus, pertussis, hepatitis B, and *Haemophilus influenzae* type b) in a study conducted in Brazil between 2020 and 2022 ^12^. An analysis of the disproportionality of notifications of events supposedly attributable to vaccination against COVID-19 in children and adolescents identified events not described in the package insert, based on Brazilian data from 2022 ^13^.

The studies that responded to the call of RESS, in this supplement issue alluding to the 25^th^ anniversary of EpiSUS, show the application of epidemiological methods in real situations of the SUS services, with the production of valuable health surveillance information. The broad scope of themes, designs, and contexts analyzed reflects the insertion of EpiSUS at the different levels of the SUS and contributes to the qualification of the response to events of public health interest. By contributing to the dissemination of this body of evidence, RESS reaffirms the importance of in-service training and field epidemiology for the strengthening of health surveillance actions in Brazil, also reinforcing its role as journal of SUS.
