# Supplementary material for: Twenty five years of the Training Program in Epidemiology Applied to the Unified Health System Services: contributions of field epidemiology to health surveillance in Brazil
Source: Epidemiol Serv Saude. 2026 May 12;35:e20263002. doi: 10.1590/S2237-96222026v35e20263002.en (PMC13686366; doi:10.1590/S2237-96222026v35e20263002.en)
Supplement: Translation

# Translation: Vinte e cinco anos do Programa de Treinamento em Epidemiologia Aplicada aos Serviços do Sistema Único de Saúde: contribuições da epidemiologia de campo para a vigilância em saúde no Brasil

**DOI:** 10.1590/S2237-96222026v35e20263002.pt

**Authors:** Mariana Del Grossi Moura, Taís Freire Galvão, Maria Auxiliadora Parreiras Martins, Everton Nunes da Silva, Jorge Otávio Maia Barreto

**Affiliations:** 1 Universidade de Sorocaba, Programa de Pós-graduação em Ciências Farmacêuticas, Sorocaba, SP, Brasil; 2 Universidade Estadual de Campinas, Faculdade de Ciências Farmacêuticas, Campinas, SP, Brasil; 3 Universidade Federal de Minas Gerais, Faculdade de Farmácia, Belo Horizonte, MG, Brasil; 4 Universidade de Brasília, Faculdade de Ciências e Tecnologias em Saúde, Brasília, DF, Brasil; 5 Fundação Oswaldo Cruz, Gerência Regional de Brasília, Brasília, DF, Brasil

A vigilância em saúde constitui função essencial do Sistema Único de Saúde (SUS), com caráter transversal e orientador do modelo de atenção. Envolve coleta, análise e disseminação de informações sobre a situação de saúde, com vistas ao planejamento e à implementação de ações de promoção, proteção e prevenção, bem como ao controle de riscos, doenças e agravos ^1^. Nesse contexto, a qualificação de profissionais para atuação nos serviços integra as ações necessárias ao desenvolvimento e à organização dessas atividades no SUS.

O Programa de Treinamento em Epidemiologia Aplicada aos Serviços do Sistema Único de Saúde (EpiSUS), da Secretaria de Vigilância em Saúde e Ambiente do Ministério da Saúde (SVSA/MS), insere-se nesse contexto como estratégia de formação em serviço, com ênfase na aplicação de métodos epidemiológicos no cotidiano dos serviços de saúde ^2^. Estruturado em diferentes níveis de formação, busca desenvolver competências para detecção, investigação e resposta a eventos de interesse em saúde pública, articulando atividades práticas e supervisão técnica. Desde sua implementação, tem contribuído para a qualificação de profissionais do SUS e para a realização de investigações de campo e análises epidemiológicas voltadas às demandas da vigilância em saúde ^2^.

Em comemoração aos 25 anos do EpiSUS, em 2025, a *Epidemiologia e Serviços de Saúde: revista do SUS* (RESS), com apoio do Departamento de Emergências em Saúde Pública da SVSA/MS, abriu uma chamada para publicação de pesquisas de campo elaboradas no contexto do programa. Além de celebrar essa trajetória do programa, a RESS buscou contribuir para a disseminação de evidências resultantes das investigações conduzidas por seus egressos. Como resultado, foram publicados 12 artigos originais, que refletem contribuições relevantes ao SUS na última década, e constituem o objeto deste editorial.

No artigo inicial, é relatada a experiência de implementação do EpiSUS entre 2000 e 2025, com apresentação dos marcos históricos e expansão dos níveis de formação, descrevendo-se a qualificação de mais de 4 mil profissionais em epidemiologia de campo ^2^.

Investigações de surtos e outros eventos epidemiológicos agudos, importantes motivadores para o estabelecimento do programa, foram retratados em diferentes abordagens. Estudo de coorte retrospectivo descreveu surto de infecções por *Mycobacterium abscessus* associado a cirurgias estéticas em região de fronteira com o Paraguai, no período de 2021 a 2022, com maior risco de adoecimento em procedimentos realizados entre dezembro e janeiro e em casos de lipoabdominoplastia ^3^. A descrição de um surto de varicela em território indígena na Bahia, em 2024, identificou cobertura vacinal abaixo da meta preconizada e descreveu as medidas de controle adotadas, incluindo atualização vacinal e busca ativa de casos ^4^. Um artigo descreveu o perfil clínico-epidemiológico e as exposições dos primeiros casos autóctones de febre do Oropouche na região Sul do Brasil, entre abril e junho de 2024, em área previamente não endêmica, com ausência de histórico de deslocamento para outras Unidades da Federação ou ao exterior, além de identificar exposições ambientais compatíveis com a transmissão pelo vetor, como corpos d’água e áreas com vegetação natural ^5^. 

Um surto alimentar de hepatite A ocorrido em Curitiba entre 2023 e 2024 resultou em cinco contribuições que elucidaram diferentes aspectos da investigação de campo. Um estudo caso-controle identificou maior chance de hepatite A associada ao consumo de peixe cru e a homens que fazem sexo com homens, enquanto a realização de refeições em domicílio foi fator protetor ^6^. Entre adolescentes, observou-se baixa soroprevalência de anticorpos de longa duração contra hepatite A, com predominância de imunidade vacinal ^7^. Nessa mesma população, observaram-se baixos níveis de conhecimento sobre a doença, especialmente quanto à transmissão e aos sintomas, além de práticas sexuais de risco ^8^. Em pessoas em situação de rua, em uma série de casos, foi observada maior frequência de formas graves e maior letalidade, em comparação à população geral, evidenciando a vulnerabilidade desse grupo no contexto do surto ^9^. A partir de outra série de casos, foi descrita a ocorrência de formas graves da doença, incluindo óbitos e necessidade de transplante hepático, além de complicações clínicas relevantes e necessidade de suporte intensivo ^10^.

Estudos sobre imunizações integram os temas abordados nesta edição. Um estudo transversal realizado em São Sebastião, no Distrito Federal, estimou cobertura vacinal contra febre amarela de 54%, inferior à meta estabelecida pelo Programa Nacional de Imunizações, e descreveu conhecimentos, atitudes e práticas da população residente, verificando-se que a maioria teve conhecimento inadequado sobre a vacinação contra febre amarela, mas com maior frequência de atitudes e práticas adequadas ^11^. 

Análise de eventos supostamente atribuíveis à vacinação, com base nos critérios de causalidade, identificou associação entre eventos graves e características como lote, apresentação e laboratório produtor da vacina pentavalente, que protege contra difteria, tétano, coqueluche, hepatite B e *Haemophilus influenzae* tipo b, em estudo conduzido no Brasil entre 2020 e 2022 ^12^. Análise de desproporcionalidade de notificações de eventos supostamente atribuíveis à vacinação contra covid-19 em crianças e adolescentes identificou eventos não descritos em bula, com base em dados do Brasil em 2022 ^13^.

Os estudos que atenderam ao chamado da RESS, em suplemento alusivo aos 25 anos do EpiSUS, evidenciam a aplicação de métodos epidemiológicos em situações reais dos serviços, com produção de informações relevantes para a vigilância em saúde. A diversidade de temas, delineamentos e contextos analisados reflete a inserção do programa nos diferentes níveis do SUS e contribui para a qualificação da resposta a eventos de interesse em saúde pública. Ao contribuir para a disseminação desse corpo de evidências, a RESS reafirma a importância da formação em serviço e da epidemiologia de campo para o fortalecimento das ações de vigilância em saúde no Brasil, reforçando também seu papel como revista do SUS.
